# Analog and RF performance optimization for gate all around tunnel FET using broken-gap material

**DOI:** 10.1038/s41598-022-22485-6

**Published:** 2022-10-29

**Authors:** Pankaj Kumar, Kalyan Koley, Bhubon C. Mech, Ashish Maurya, Subindu Kumar

**Affiliations:** 1 Department of Electronics Engineering, Indian Institutes of Technology, Dhanbad, Dhanbad, 826004 India; 2 Department of Electronics and Communication Engineering, Birla Institute of Technology, Mesra, Ranchi, 835215 India; 3 Department of Electronics and Communication Engineering, Defence Institute of Advanced Technology, Girinagar, Pune, 411025 India

**Keywords:** Engineering, Materials science, Nanoscience and technology

## Abstract

Many times, the fabricated cylindrical gate-all-around tunnel FET (GAA TFET) has an uneven radius due to several etching and deposition processes involved while fabricating the device, which show notable variations in the performance of the device. In this report, III–V uneven GAA TFET is studied by considering the uneven radius as elliptical in shape for all possible variations, which shows a significant impact on analog and RF figure of merits (FOMs). The performance of the optimized devices is compared with their circular structure and with their maximum deviation in elliptical geometry for all possible variations in device channel and gate oxide. The variations in its device channel and gate oxide have shown a significant impact on the performance of the device. The analog and RF FOMs are studied, including the transconductance generation factor (g_m_/I_DS_), intrinsic gain (g_m_R_O_), capacitances (C_GS_, C_GD_), cut-off frequency (f_T_), and gate delay (τ_m_).

## Introduction

Aggressive transistor scaling with the aim of boosting on-die capabilities has led to an adverse effect on power dissipation. Due to increasing insistence on power dissipation, low voltage requirements, energy conservation, and maximising on-die capabilities has led the devices having the prospect of acquiring sub-threshold swing (SS) surpassing the thermionic limit of 60 mV*/*dec has earned extensive attention^1–3^. Tunnel field-effect transistors (TFETs) have unique carrier injection mechanisms and inversion layer formation when compared to conventional MOSFETs. These lead to substantial improvement of SS, I_*ON*_/I_*OFF*_ ratio, and, as such, TFET devices have been one of the prominent contenders for replacing conventional MOSFETs^4,5^. However, TFETs have encountered severe ambipolar leakage current (I_*AMB*_), low ON-state current (I_*ON*_), and gradual shifting between ON and OFF states^6^. With continuous scaling of devices, the reduction of power dissipation remains a primary requisite along with the enhancement of device performance as far as system-on-chip based CMOS integrated circuits are concerned. In addition, the gate-all-around (GAA) architecture has demonstrated enhanced electrostatic control, tunneling efficiency, off-state leakage current, and current drivability, thereby, resulting in the improvement of analog and RF performances^7–9^.

The steps involved in the fabrication of the vertical GAA structure involve several etching, deposition, and growing processes, which generally form an uneven radius rather than the ideal circular GAA structure^10,11^. Because of this uneven radius, predicting the performance of fabricated GAA device structures is difficult^12,13^, and only a few reports for its acceptable variations^14–17^ are available. Most of the reports only address one type of geometry and are of mostly MOSFET devices, as the report^12^ shows the impact of hydrogen annealing on the device diameter of GAA MOSFET devices, which led to the elliptical circumference having an ON current of 825 µA/µm, report^13^ shows the fabricated GAA MOSFET device having an elliptical shape (i.e., EOEC geometry) having ON current of 976 µA/µm, report^15^ demonstrate the impact of varying channel length on fabricated elliptical GAA MOSFETs, report^14^ demonstrates numerical analysis on three different types of variations such as t_si_ fluctuation, elliptical shape fluctuations (i.e., EOEC fluctuations), and corner rounding fluctuations where the acceptable tolerance for RF IC design is analyzed as Δt_si_ < 1 nm and r/R > 75%, and the report^16^ mathematically analyzed the short-channel-effects of elliptical GAA MOSFET (i.e., EOEC structure) by varying its effective radius. The experimental^18,19^ and numerical analyses^20^ suggest that incorporating III-V broken-bandgap semiconductor material in TFET devices shows improved tunnelling efficiency, leading to enhanced device performance^21,22^. The ternary compound material of type-II heterostructure such as GaSb/InGaAs gained importance due to its tunable band alignment, direct bandgap, and effective bandgap by varying the mole fraction^23–25^. There are some challenges while fabricating III–V semiconductor TFETs, such as a lack of dielectric material, which causes thermodynamically unstable interface states, thereby leading to fermi level pinning at the interface. Moreover, uncontrolled oxidation on III–V material results in a higher density of bandgap traps at the interface. However, these challenges are encountered by using the interface control (i.e., passivation) layer at the interface^26^.

In this report, the issues related to fabricating vertical GAA TFET are considered where the most unlikely uneven radius is considered to be elliptical in shape for all possible GAA fabrication defects. In addition, a broken-gap type-II heterojunction of GaSb/InGaAs material has been introduced^23^, which further boosts the device performance. The objective of this paper is to design and optimize III–V elliptical gate-all-around TFET (III–V eGAA TFET) by varying the minor diameter (MD) and gate dielectric thickness (T_OX_) of the channel region to get the optimized MD and T_OX_ of the elliptical structure. The optimization process is carried out by considering the higher I_*ON*_, lower I_*AMB*_, low parasitic capacitance, minimum gate delay (τ_m_), and higher mobility. Finally, the optimal structure is investigated for analog and RF performance.

### Device structure and simulation methodology

**Figure 1:**
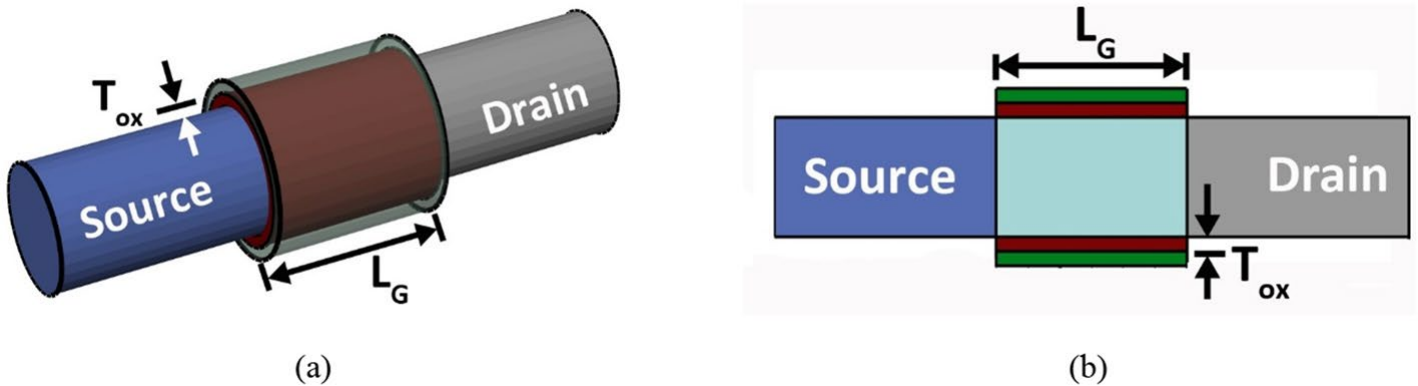
EOEC eGAA TFET structure: (**a**) 3D view, (**b**) cross-sectional view.

**Figure 2:**
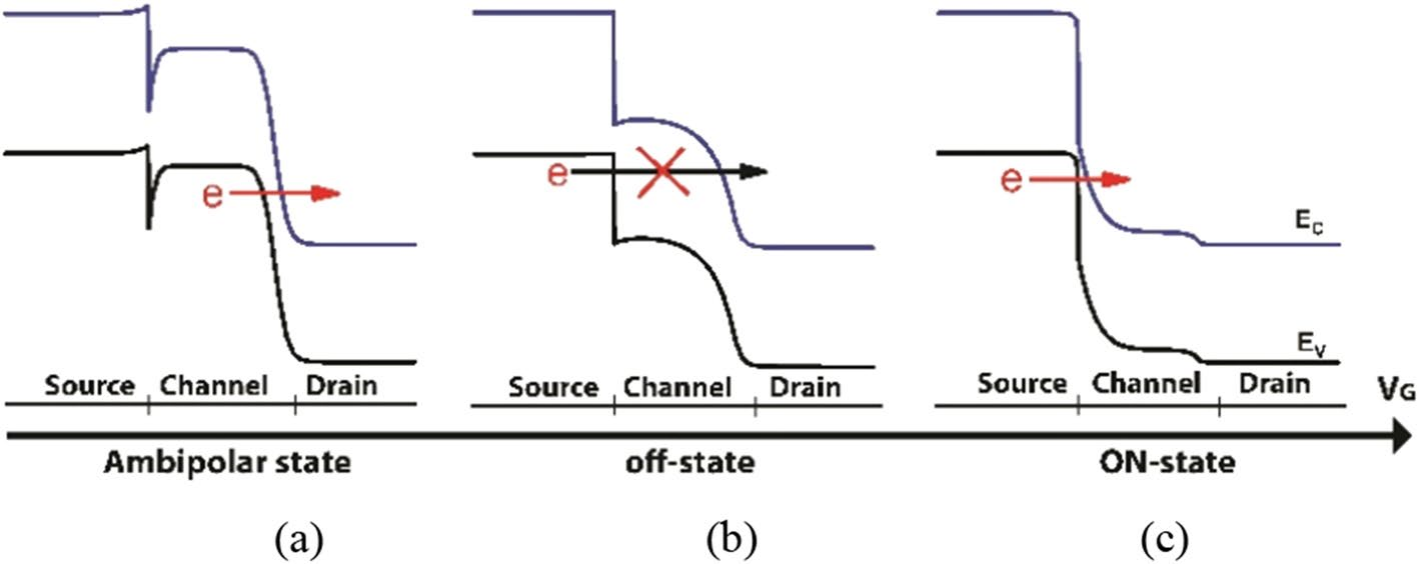
Band diagram of COCC structure: (**a**) ambipolar-state, (**b**) off-state, (**c**) ON-state.

The 3D and cross-sectional schematics of the simulated III–V eGAA TFET structure for elliptical oxide–elliptical channel (EOEC) is shown in Fig. 1, and its corresponding band diagram for circular oxide–circular channel (COCC) for ambipolar, off, and ON state conditions are depicted in Fig. 2. The device under simulation incorporates type-II heterojunction material such as GaSb/InGaAs^23^. In addition, the uneven radius of the fabricated device is considered elliptical in nature, having a major diameter of 20 nm, and the optimal value of the minor diameter (MD) is calibrated by varying it from 10 to 20 nm. The device also incorporates the source doping (*N*_*A*_) of 10^20^ cm^−3^, intrinsic channel doping (N_*I*_) of 10^15^ cm^−3^, drain doping (N_*D*_) of 5 × 10^19^ cm^−3^, gate length (L_G_) of 30 nm, and maximum gate oxide thickness (T_OX_) of 2 nm. The inset of our simulation is in agreement with ITRS^27^ and the targeted value considered in our simulation is tabulated in Table 1, where the work function of the metal and supply voltage (V_DD_) are set at 5.01 eV (i.e., nickel) and 0.3 V*,* respectively. The simulations are carried out by the *3D* numerical device simulator TCAD Sentaurus^28^ with an optimized meshing strategy as discussed in^29^. The non-local band-to-band tunnelling (BTBT) model has been employed in our simulation. Apart from this, carrier scattering due to the presence of charged impurity ions and velocity saturation at high-field is activated by considering concentration-dependent and high-electric field-dependent mobility models. The mobility degradation model at interfaces is also included to compute the transverse field, and the old Slotboom model is incorporated to compute the doping-induced bandgap narrowing of the material. Moreover, the effect of the strain through deformation in the lattice structure, causing shifts in the conduction and valance bands, is captured by the piezo deformation potential model. Finally, the quantum correction model is incorporated to take into account the change in the effective bandgap of the device due to the variation in MD from 10 to 20 nm. The non-local BTBT parameters of Sentaurus TCAD for InGaAs and GaSb are used as described in^30^.

**Table 1:** ITRS 2013 (predicted LP technology requirements of multi-gate (MG) FET devices for year 2021).

Parameter	Predicted value for year 2021	Obtained value
V_DD_ for III–V material, (V)	0.59	0.3
C_GG_ for III–V material, (fF/µm)	0.59	0.0385
Mobility, (cm^2^/V-s)	300	15,509.8
*I*_*OFF*_ (pA/µm)	20	6.7

### Proposed fabrication steps

**Figure 3:**
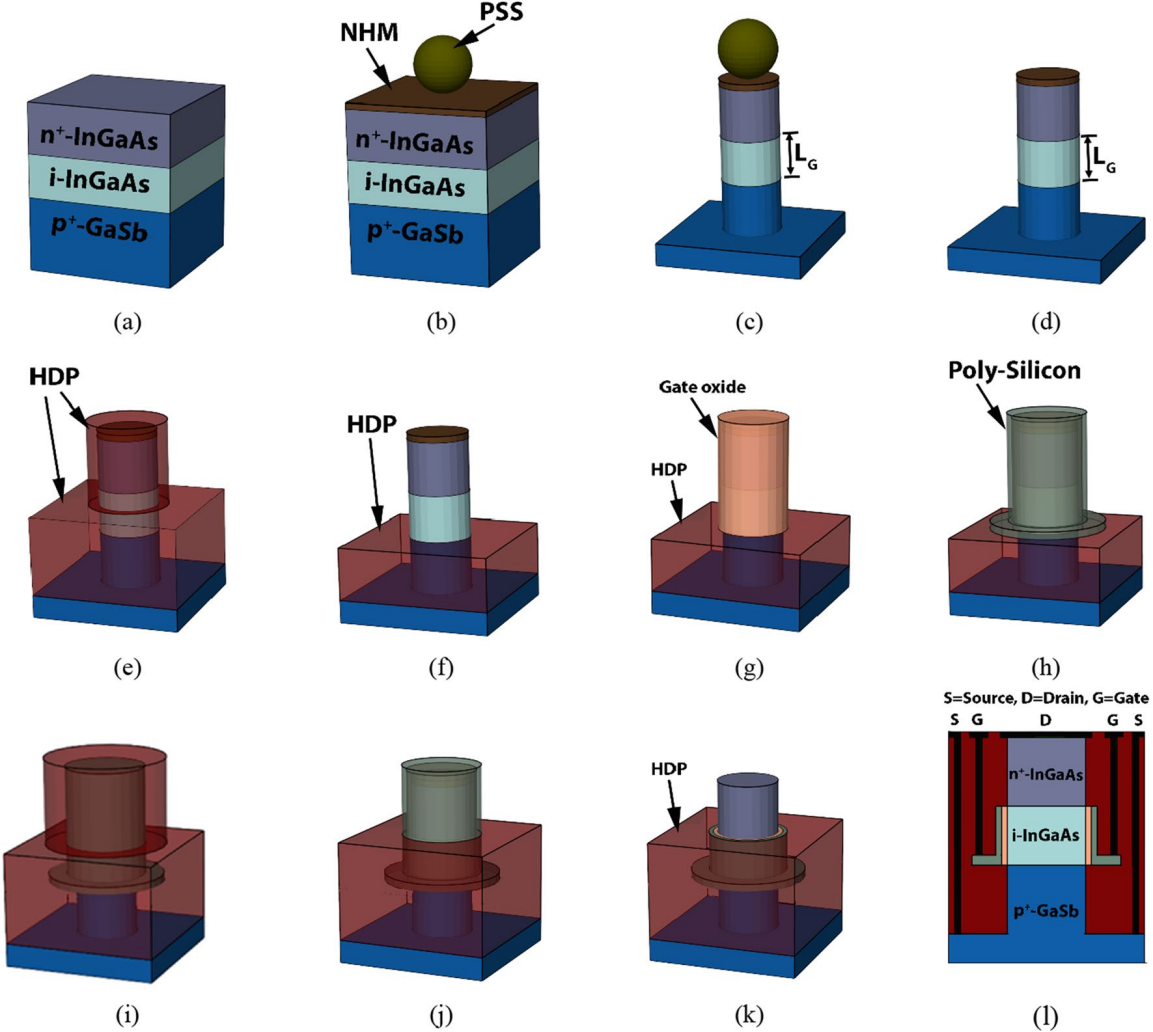
Schematic of III–V eGAA TFET fabrication steps: (**a**) III–V material deposition, (**b**) PSS placement, (**c**) RIE on PSS to get 20 nm sphere followed by NHM deposition, (**d**) deep RIE etching process, (**e**) PSS of 20 nm diameter and NHM etched away, (**f**) SiO_2_ deposition, (**g**) PSS of 24 nm diameter and NHM deposition, (**h**) DHF etch-back up to the source-channel junction, (**i**) PolySilicon deposition, (**j**) SiO_2_ deposition, (**k**) contact tip implantation followed by contact formation.

The process steps for fabricating III–V eGAA TFETs are proposed in Fig. 3. The sequential process steps which can be employed for the fabrication are: (i) Layers of GaSb (p-type, Be ~ 10^20^ cm^−3^), 30 nm of InGaAs (intrinsic, Si ~ 10^15^ cm^−3^), and 35 nm of InGaAs (n-type, Si ~ 5 × 10^19^ cm^−3^) need to be deposited layer-by-layer as shown in Fig. 3a. Such a deposition can be carried out by molecular beam epitaxy (MBE). Then, a nitride hard mask (NHM) is deposited on top of the surface followed by a spreading polystyrene sphere (PSS) to self-assemble on top of the surface (Fig. 3b). To reduce the PSS diameter, the reactive ion etching (RIE) process can be used, which forms colloidal particle nanodots that are no longer closely packed (i.e., space is created for gate oxide and contact pad deposition) for getting the GAA structure. (ii) Tetramethylammonium hydroxide (TMAH) based wet etch process needs to be carried out to remove the material underneath the NHM patterned by PSS (Fig. 3c). The next step involves etching away the PSS with the dissolution of CHCl_3_ (Fig. 3d).

Further, a non-conformal high-density plasma (HDP) oxide is deposited (Fig. 3e), followed by dilute hydrofluoric acid (DHF) etch-back to get HDP up to the source-channel junction (Fig. 3f). (iii) To grow gate oxide high-density plasma chemical vapor deposition (HDPCVD) technique can be used (Fig. 3g), followed by the deposition of poly-silicon (Fig. 3h). (iv) Again, non-conformal HDP oxide is deposited (Fig. 3i) and DHF etch-back to get HDP up to the channel-drain junction (Fig. 3j). Further, etch-back the poly-silicon, gate oxide, and NHM to expose the drain region (Fig. 3k). Finally, non-conformal HDP oxide is deposited again and patterned to form the contact pads (Fig. 3l).

**Figure 4:**
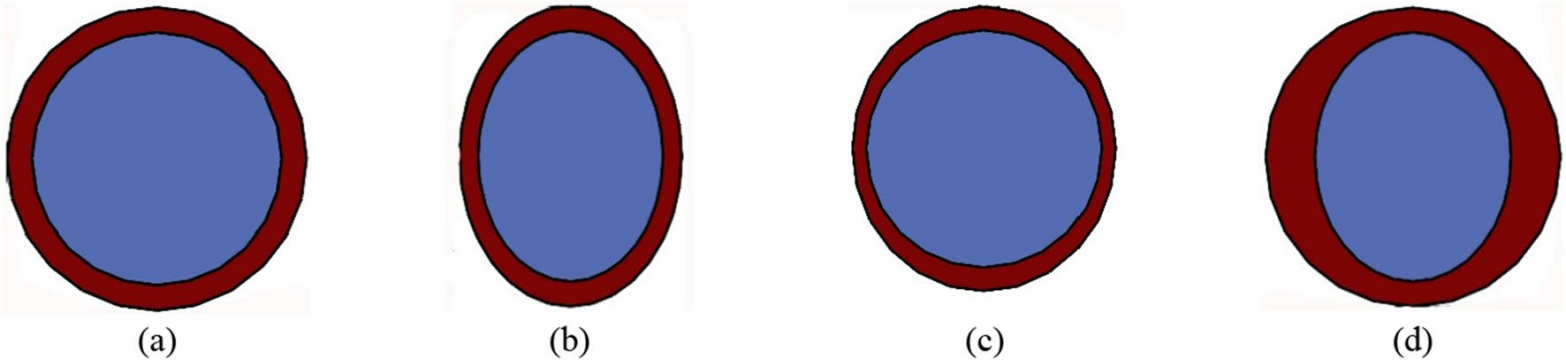
Schematic representation of possible cross-sectional variations of GAA structure due to fabrication imperfections: (**a**) circular oxide—circular channel (COCC), (**b**) elliptical oxide—elliptical channel (EOEC), (**c**) elliptical oxide—circular channel (EOCC), (**d**) circular oxide—elliptical channel (COEC).

We have considered that the RIE process is involved in reducing the PSS and the grown gate oxide, which accounts for the uneven channel radius and T_OX_ variation induced in the fabricated device. The worst-case scenario of structural variations involved in the fabrication process is considered elliptical in shape as depicted in Fig. 4, where, Fig. 4a shows the ideal circular GAA structure having a circular oxide-circular channel (COCC). Figure 4b to Fig. 3d represent elliptical oxide—elliptical channel (EOEC), elliptical oxide—circular channel (EOCC), and circular oxide—elliptical channel (COEC), respectively.

### Structural optimization

Device optimization for EOEC and COEC structure is carried out by evaluating optimal channel MD by varying it from 10 to 20 nm. For the EOCC structure, optimization is done by varying T_OX_ from 1 to 2 nm along the minor axis (MA).

**Figure 5:**
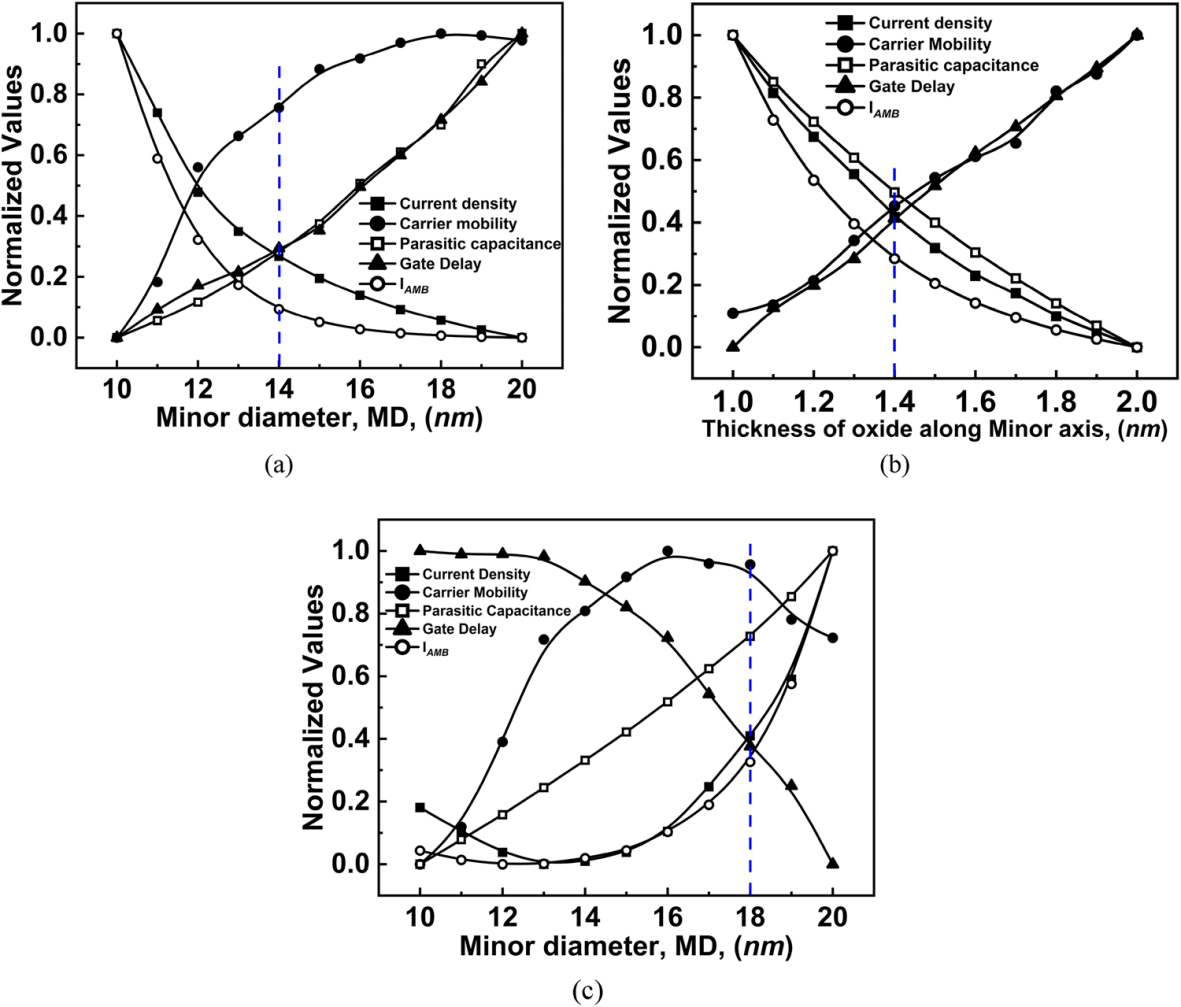
Normalized values for different eGAA TFET along: (**a**) MD for EOEC optimization, (**b**) MA of EOCC structure for gate oxide thickness optimization, (**c**) MD for COEC optimization.

**Figure 6:**
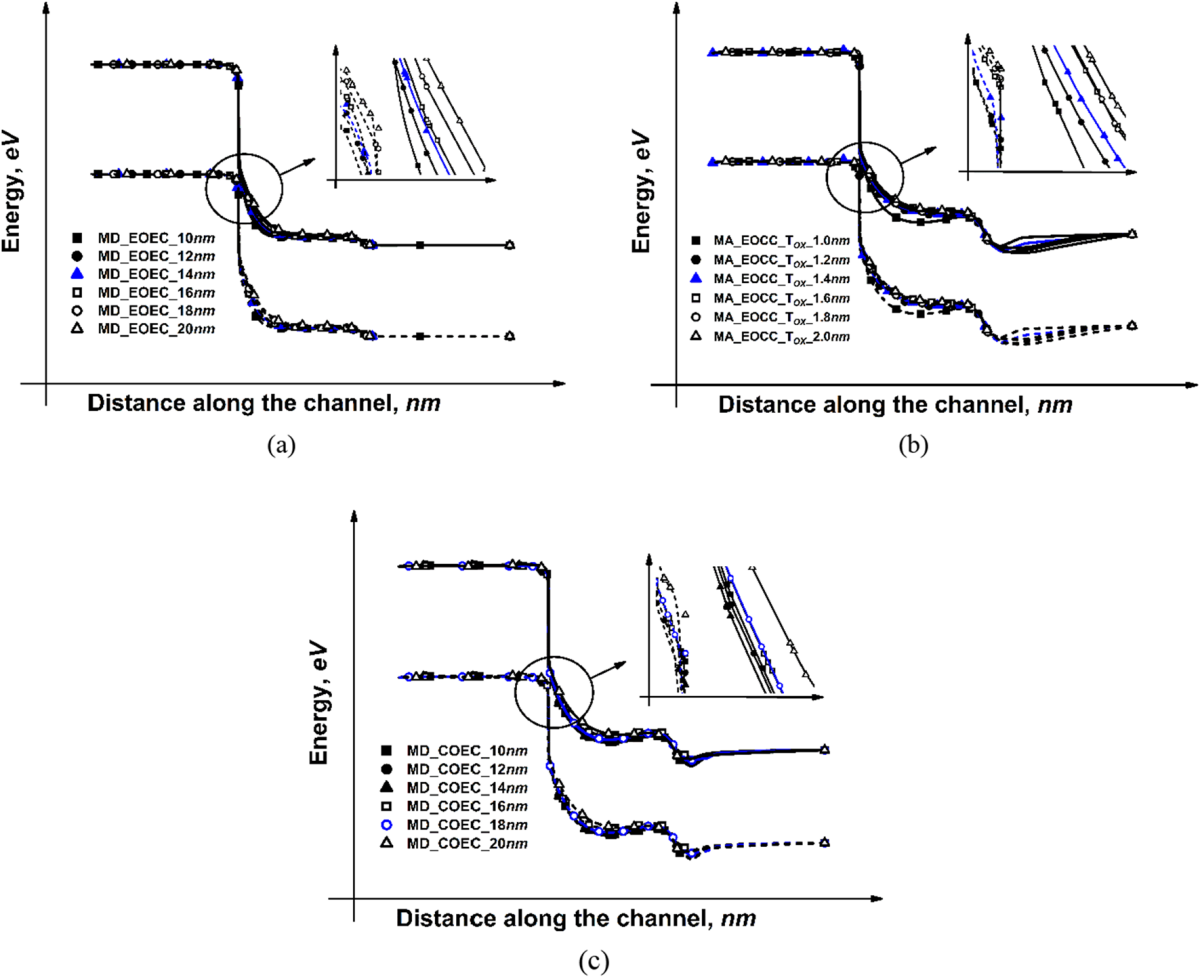
Band diagram for different eGAA TFET along: (**a**) MD for EOEC optimization, (**b**) MA of EOCC structure for gate oxide thickness optimization, (**c**) MD for COEC optimization.

For optimization of the EOEC structure, the major diameter of the channel region is fixed at 20 nm, while the MD is decreased from 20 nm (which represents a circular GAA TFET) to 10 nm (eGAA TFET), simultaneously maintaining the same gate oxide thickness of 2 nm throughout the entire elliptical periphery. The results of our simulation are depicted in Fig. 5a. Starting from 20 nm, as the channel, MD is reduced, the band bending between the drain-channel and source-channel region gradually increases as depicted in Fig. 6a. The former increases the inflow of carriers from drain to channel, increasing I_*AMB*_ of the device; the latter increases the inflow of carriers from source to channel, increasing J_DS_ of the device. As the channel MD is reduced, the area of the device decreases gradually, which consequently decreases C_par_ followed by a decrease in its τ_m_. For EOEC structure optimization, the point at which the structure has maximum J_DS_ and µ_carr_, and the minimum possible I_*AMB*_, C_par_, and τ_m_ is considered to be the optimized geometry. In this report, an EOEC structure having a MD of 14 nm is considered to be the optimized device.

For EOCC structural optimization, the channel region is fixed at 20 nm in diameter from all sides, while the T_OX_ is reduced from 2 nm (which represents circular GAA TFET) to 1 nm (eGAA TFET) along MA, while keeping a fixed T_OX_ of 2 nm along the major axis. The results of our simulation are depicted in Fig. 5b. Starting from 2 nm, as the T_OX_ along MA is reduced, the vertical electric field penetrates more into the substrate along MA, which consequently increases J_*DS,*_ and the same is verified from the energy band diagram depicted in Fig. 6b where tunneling width becomes narrow with reducing T_OX_ along MA. As the vertical electric field is penetrating more into the substrate causes a narrowing of depletion width at the drain to the channel region. The reduced depletion width at the drain to channel region gives rise to the inflow of carriers from drain to channel, which leads to an increase in I_*AMB*_, followed by an increase in its C_par_. However, τ_m_ depends on J_*DS*_ and the total gate capacitance of the device, where J_*DS*_ shows the dominant factor which leads to a decrease in its τ_m_. For EOCC structure optimization, the point at which the structure has maximum J_DS_ and µ_carr_, and the minimum possible I_*AMB*_, C_par_, and τ_m_ is considered to be the optimized geometry. In this report, the EOCC structure having a T_OX_ of 1.4 nm along MA is considered to be the optimized device.

Finally, for COEC structural optimization, the outer diameter (both channel and T_OX_ together) is fixed at 24 nm from all sides, while with a decrease in channel MD from 20 to 10 nm (all are having an outer diameter of 24 nm) lead to virtual increment of T_OX_ from 2 to 7 nm along MD of the device as depicted in Fig. 4d. Result of our simulation depicted in Fig. 5c does not explicitly show the effect of variation of neither channel MD nor T_OX_ characteristics as obtained in Fig. 5a,b. For COEC characteristics, the impact of channel MD is observed up to certain channel MD, thereafter the impact of T_OX_ plays a significant role as depicted in Fig. 5c. Starting from 20 nm, as the channel, MD is reduced, the J_*DS*_ decreases up to 14 nm considering the same reason stated for Fig. 5a, thereafter J_*DS*_ increases due to the impact of T_OX_ which becomes more dominant than the impact of channel MD and follows the same reason stated for Fig. 5b^31^. The variation in current density is also verified by the energy band diagram depicted in Fig. 6c. For COEC structure optimization, the point at which the structure has maximum J_DS_ and µ_carr_, and the minimum possible I_*AMB*_, C_par_, and τ_m_ is considered to be the optimized geometry. In this report, a COEC structure having a channel MD of 18 nm is considered to be the optimized device. It is observed that the channel MD of 14 nm and 18 nm for EOEC and COEC and T_OX_ of 1.4 nm for EOCC exhibit optimized device performance.

**Figure 7:**
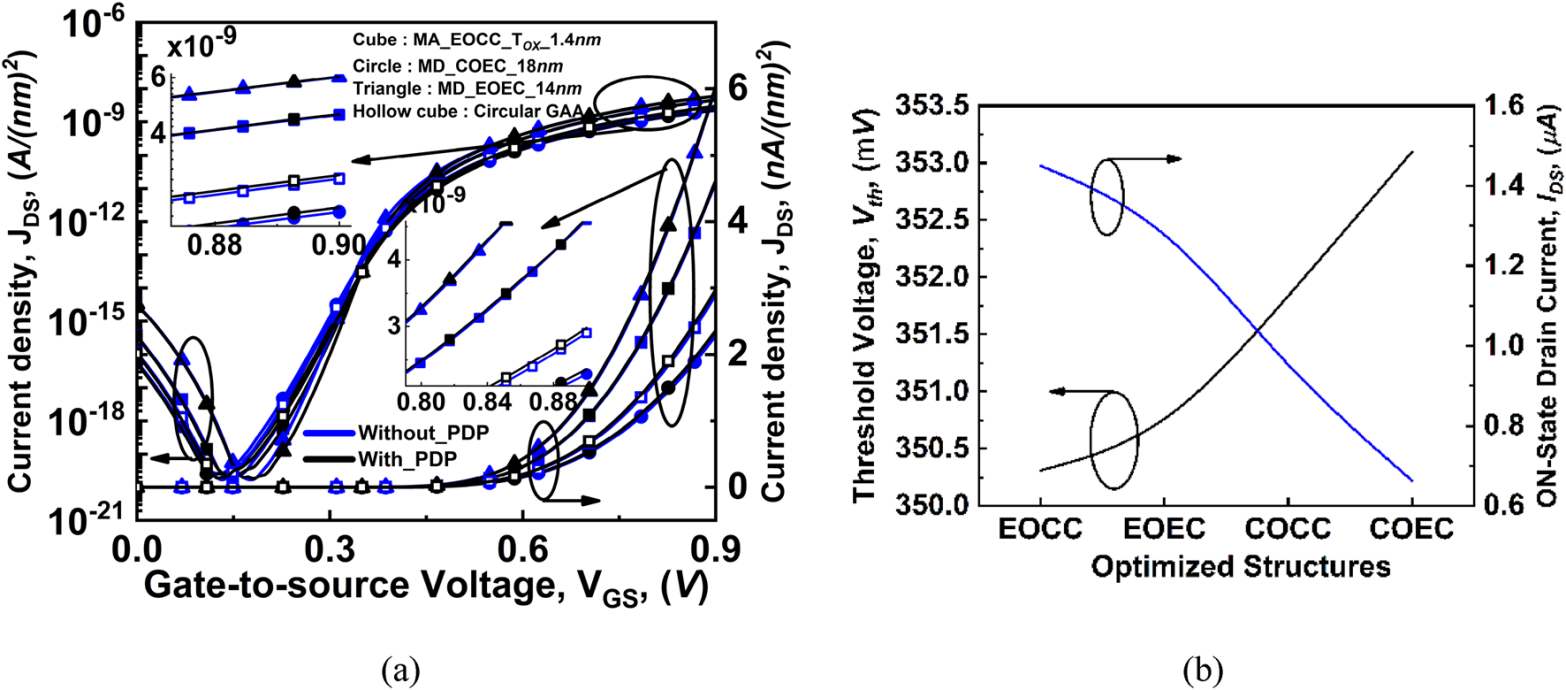
(**a**) Comparison of variation of drain current density (J_DS_) as a function of gate-to-source voltage at V_DS_ = 0.3 V for all possible optimized structures when PDP is invoked, and (**b**) variation in threshold voltage and ON-state drain current as a function of deformation in device structures.

Further, the impact of mechanical strain generated during the fabrication process, specifically at the junctions due to lattice mismatches, has been considered in the analysis. These lattice mismatches cause variation in the band structure and carrier mobility of the device, which affects the drain current, as shown in Fig. 7a. To encounter these variations, piezo deformation potential (PDP) has been invoked to get closely matched fabricated device characteristics. It is observed from Fig. 7 that, when PDP is included, then the J_*DS*_ shows an increment of ~ 3.5%, ~ 3%, ~ 0.5%, and ~ 0.4% for COCC, COEC, EOCC, and EOEC, respectively, when compared to the device which does not have PDP involved in its numerical simulation analysis. Finally, the impact of structural deformation on threshold voltage and ON-state drain current of the device is depicted in Fig. 7b. It is observed that when the channel area is large and the oxide width is comparatively thinner for the EOCC structure, then ON-state drain current is high whereas threshold voltage is comparatively low. The impact is clearly visualized when we compare EOEC and COCC structures, where oxide width is constant and channel area reduces, which causes an increase in ON-state drain current and a decrease in the threshold voltage of the device because channel area is the dominating factor which reduces current density with a reduction in the channel area. On the other hand, the impact of oxide is visualized when we compare EOCC and COEC structures, where oxide width along the minor axis is significantly high. This high oxide width causes a decrease in the device's ON-state drain current and a significant increase in the device's threshold voltage, as shown in Fig. 7b. The detailed discussion of different optimized geometries due to varying channel area and oxide width on analog and RF performance is analyzed in the result and discussion section.

## Results and discussion

This section presents the analog and RF performances of III-V eGAA TFET considering the channel MD of 14 nm and 18 nm for EOEC and COEC structures whereas T_OX_ of 1.4 nm for EOCC structures to get the better device performance at 300 ^o^K.

### Effect of process variation on analog performance parameter

**Figure 8:**
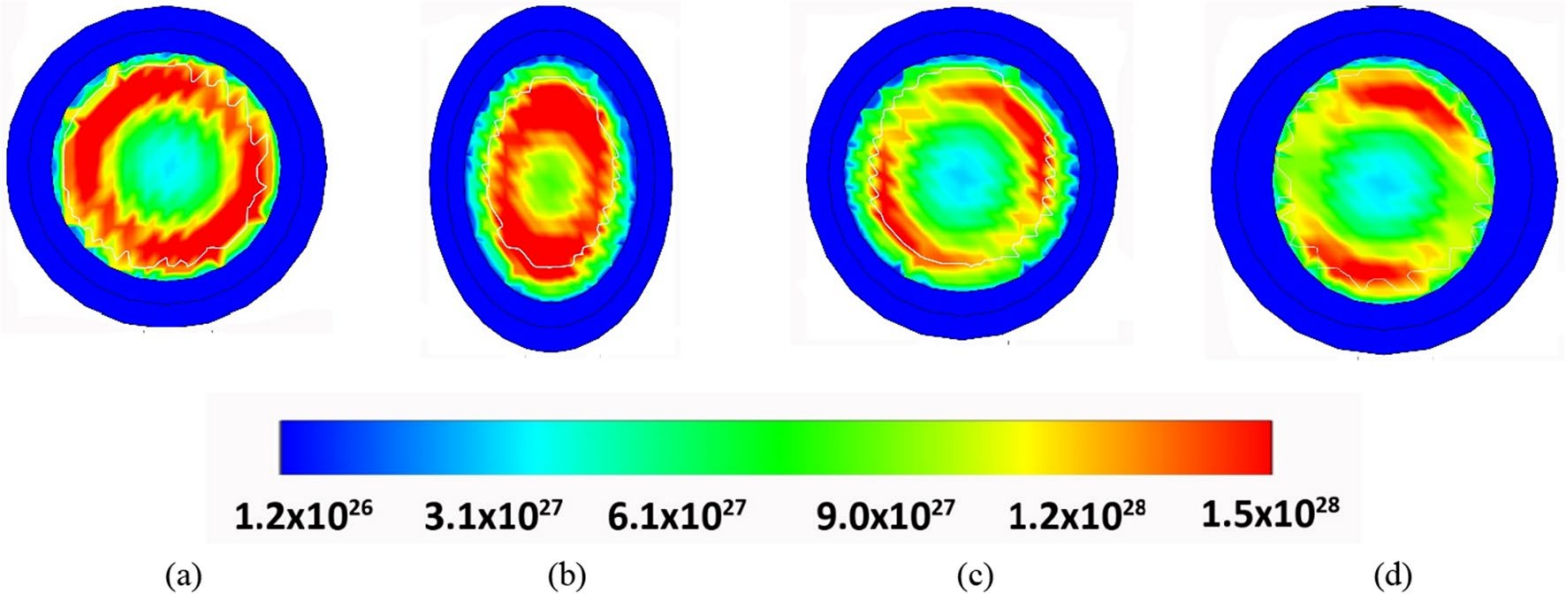
Band generation rate of carriers for (**a**) COCC, (**b**) EOEC, (**c**) EOCC, and (**d**) COEC.

The impact of channel MD and T_OX_ on the analog performance parameters such as transconductance (g_m_), output conductance (g_D_), transconductance generation factor (g_m_/I_DS_), and intrinsic gain (g_m_R_O_) for III-V eGAA TFET structure are analyzed in this section. It is observed from Fig. 8a that the COCC structure has an accumulation of band-to-band generated tunneling carriers from all sides of the gate in a circular fashion and are generally present near the semiconductor-dielectric interface. Whereas, due to the vertical electric field of EOEC structure penetrating more at the center of the device as depicted in Fig. 8b, which results in tunneling of the carrier at the center of the device rather than near the semiconductor-dielectric interface^32^. As a result, the carrier density increases at the center of the device which narrows the tunneling width at the source-channel junction as depicted in Fig. 6b^33^. Therefore, the band-to-band generated tunneling carriers take the shape of the elliptical channel and the accumulated carriers shift from the semiconductor-dielectric interface to the center of the device^34^.

**Figure 9:**
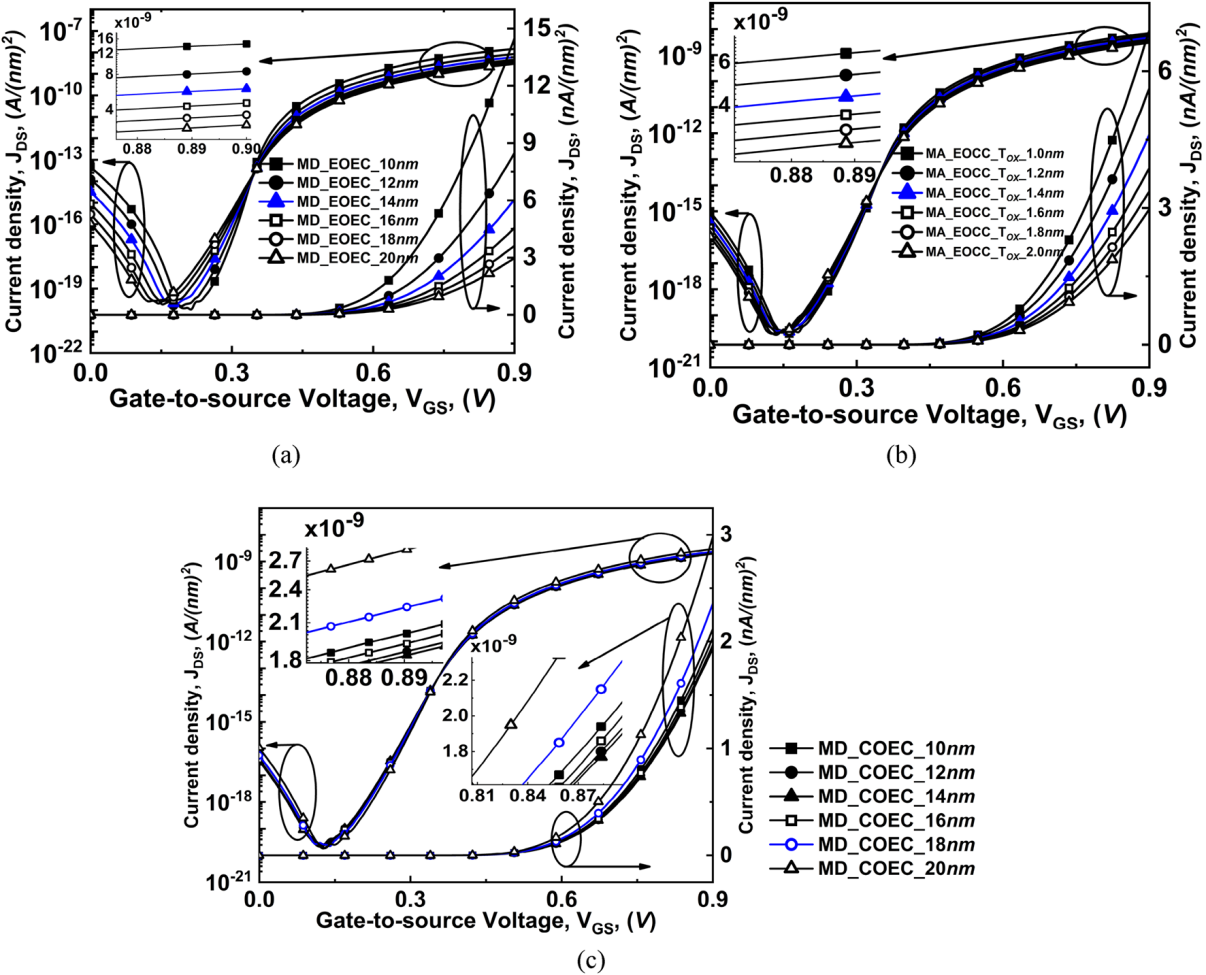
Comparison of variation of drain current density (J_DS_) as a function of gate-to-source voltage at V_DS_ = 0.3 V of COCC structure with (**a**) EOEC, (**b**) EOCC, and (**c**) COEC structure.

For EOCC structure depicted in Fig. 8c shows that the vertical electric field penetrated more into the region where T_OX_ is minimum causing carriers to accumulate at the region where T_OX_ is minimum. Finally, for COEC structure depicted in Fig. 8d, band-to-band generated carriers follow the same phenomenon stated for Fig. 8b,c considering the dominancy of either channel MD of EOEC structure or T_OX_ of EOCC structures. However, the device having channel MD of 18 nm of COEC structure shows that the carriers are accumulating below the major axis where T_OX_ is fixed at 2 nm^17,32^. The vertical electric field penetrates more with decreasing channel MD of EOEC structure resulting in an increase in its carrier density^33^ as depicted in Fig. 9a. As electron density increases cause more carriers to tunnel from the valence band of the source to the conduction band of the channel, which enhances the tunneling probability of carrier at the source to channel junction^35^. The insertion in Fig. 9a shows the increment in drain current density of optimized EOEC from ~ 3 nA/(nm)^2^ to ~ 6 nA/(nm)^2^ from its circular counterpart.

In the case of varying T_OX_ keeping fixed channel MD of 20 nm of EOCC structure causes faster accumulation of the carrier density near the semiconductor-dielectric interface where T_OX_ is minimum as depicted in Fig. 9b. However, the gradual increase in the electrostatic control of the EOCC structure shows an increment in its drain current density with a decrease in T_OX_ of the device along MA. The insertion in Fig. 9b shows the increase in the drain current density from ~ 3 nA/(nm)^2^ to ~ 5 nA/(nm)^2^ when compared to COCC structure.

On the contrary, the COEC structure shows an unpredictable trend even after keeping the outer diameter fixed at 24 nm. It is evident from Fig. 8d that, varying channel MD causes virtual variation in T_OX_ of the device which leads to accumulation of carrier density depending on the dominancy of either channel MD or T_OX_ of the device. Result of our simulation shown in Fig. 9c shows that when channel MD is 10 nm then T_OX_ along the channel MD is maximum and has a drain current density of ~ 2.2 nA/(nm)^2^ whereas with the gradual increase in channel MD from 10 to 14 nm shows the effect of T_OX_ on the device is relatively higher than the variation of MD of the channel on the device. As a result, drain current density decreases from ~ 2.2 to ~ 1.9 nA/(nm)^2^ from channel MD of 10 nm to 14 nm. However, when channel MD increases beyond 14 nm, the impact of variation of channel MD is more in the device when compared to the virtual variation of T_OX_ along MA. The enlarged view under Fig. 8c depicts that, the drain current density increases beyond 14 nm whereas the optimized COEC structure shows a drain current density of ~ 2.4 nA/(nm)^2^.

For the evaluation of the transconductance (g_m_) and the drain conductance (g_D_) of the III-V eGAA-TFETs, the following expressions are used,

1 $$g_{m} = \frac{{\partial I_{DS} }}{{\partial V_{GS} }},$$

2 $$g_{D} = \frac{{\partial I_{DS} }}{{\partial V_{DS} }}$$

The g_m_ and g_D_ expressions depend on the change in I_DS_ with changing applied port biasing of TFET^33^. However, the dependency on I_DS_ further led to a dependency on the tunneling probability of the carriers at the source-to-channel junction, effective mobility (*µ*_*eff*_)$\left({\upmu }_{\mathrm{eff}}\right)$, tunneling barrier width, or average tunneling thickness (*λ*)$\left(\uplambda \right)$ of the device.

**Figure 10:**
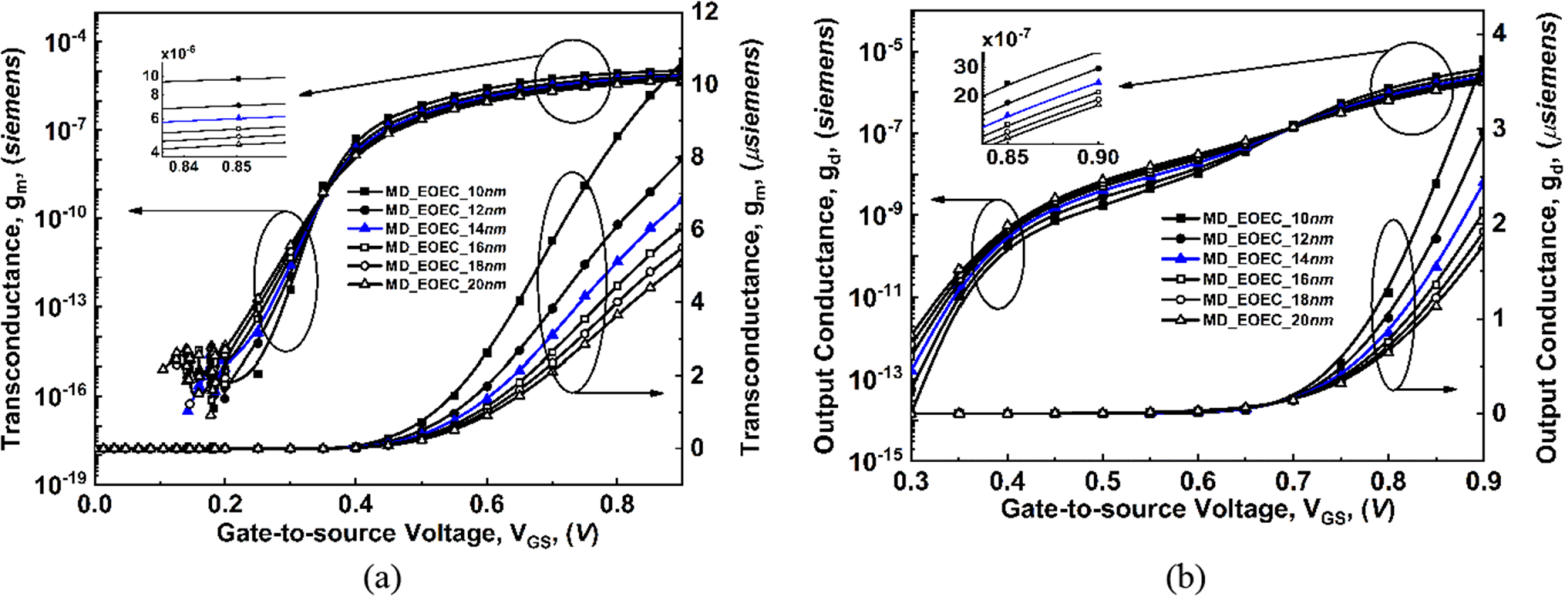
Comparison of variation of transconductance (g_m_) and output conductance (g_d_) as a function of gate voltage at V_DS_ = 0.3 V for EOEC devices: (**a**) g_m_, and (**b**) g_d_.

**Figure 11:**
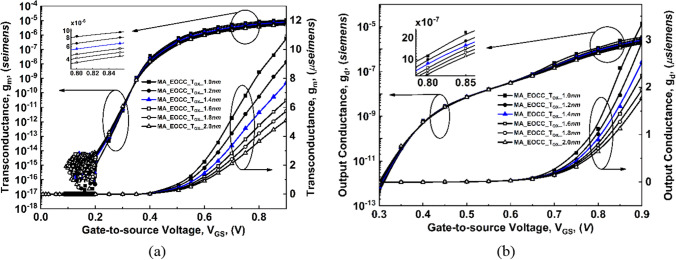
Comparison of variation of transconductance (g_m_) and output conductance (g_d_) as a function of gate voltage at V_DS_ = 0.3 V for EOCC devices: (**a**) g_m_, and (**b**) g_d_.

**Figure 12:**
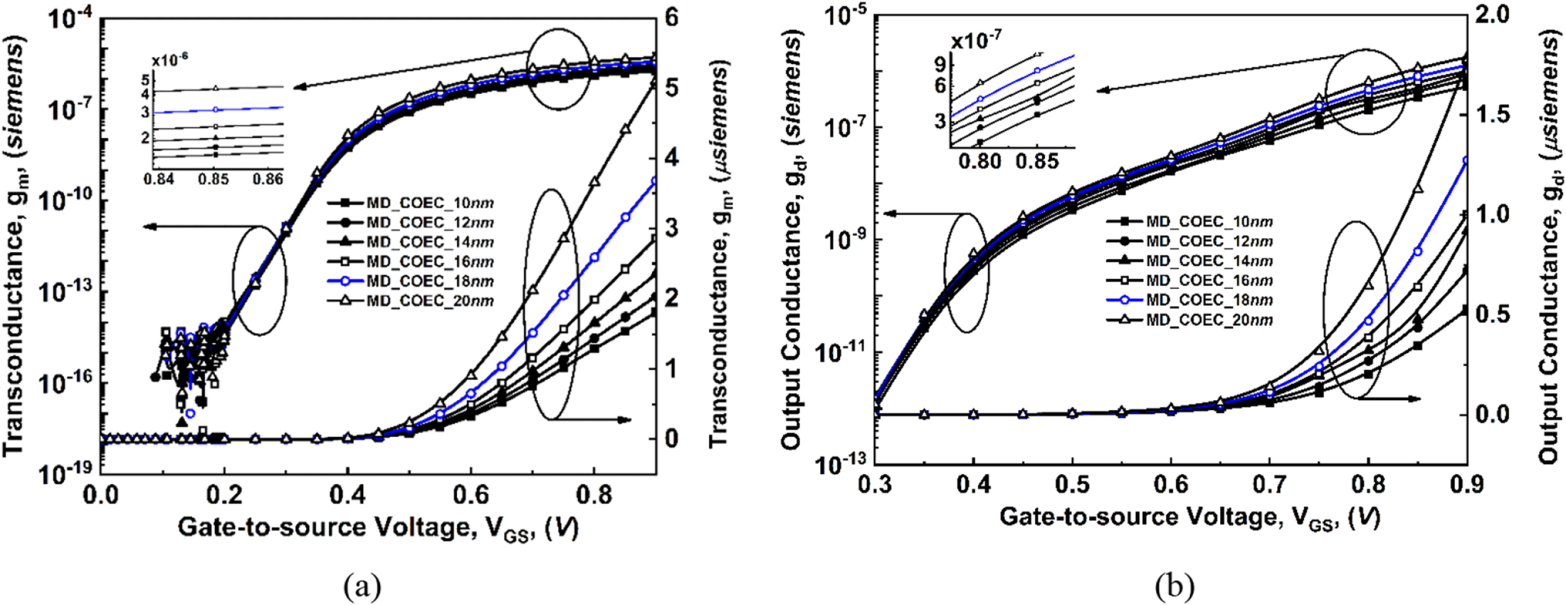
Comparison of variation of transconductance (g_m_) and output conductance (g_d_) as a function of gate voltage at V_DS_ = 0.3 V for COEC devices: (**a**) g_m_, and (**b**) g_d_.

For EOEC and EOCC structures, their tunneling barrier width becomes narrower which increases the tunneling probability, thereby, g_m_ and g_D_ show a rapid increase with V_GS_ as shown in Fig. 10 and Fig. 11. On the other hand, for COEC structure, their tunneling barrier width depends on both the variations (channel MD and T_OX_) which result in a decrease in g_m_ and g_D_ till 14 nm than a gradual increase beyond 14 nm along channel MD of the device as shown in Fig. 12.

**Figure 13:**
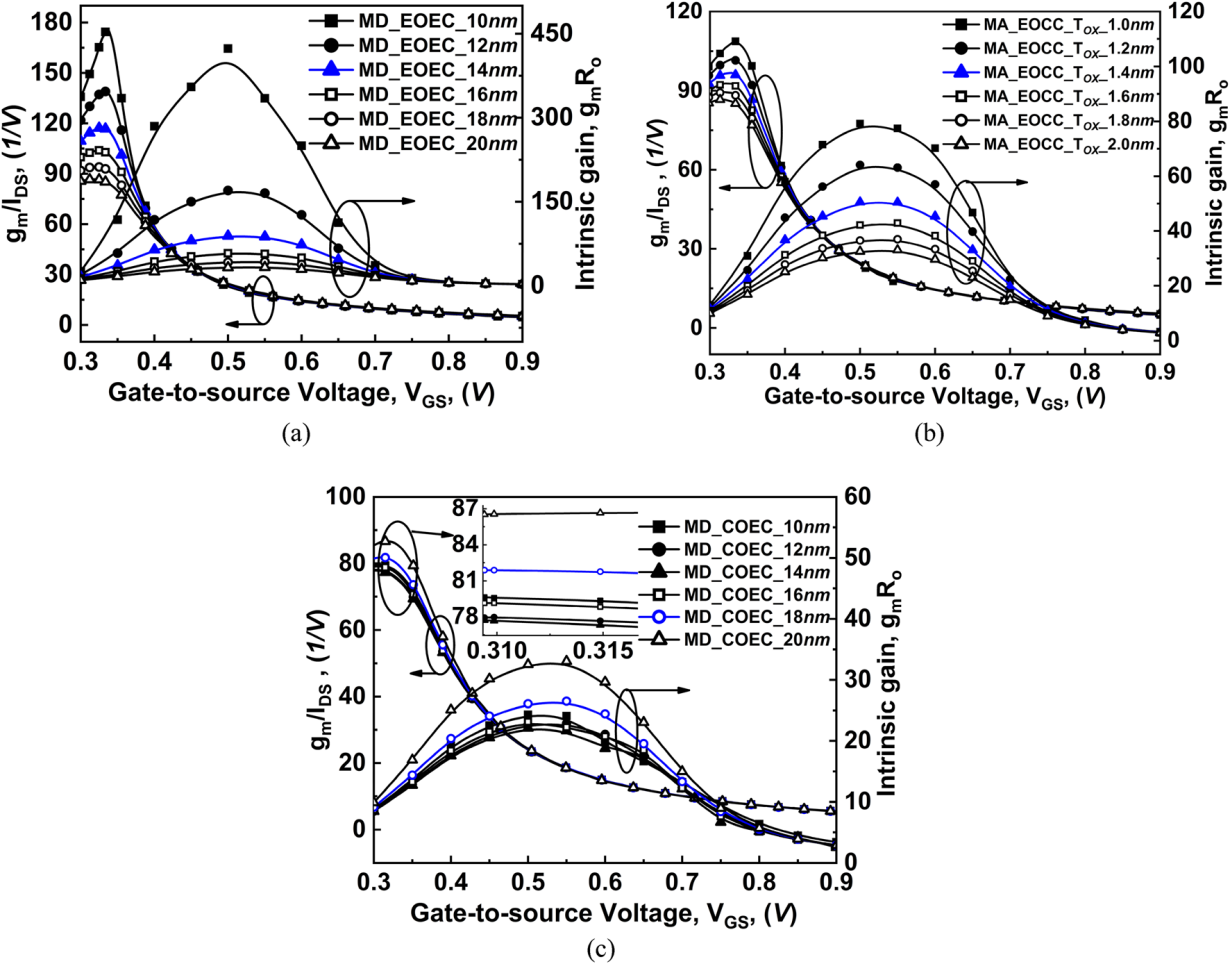
Comparison of variation of transconductance generation factor and intrinsic gain as a function of gate voltage at V_DS_ = 0.3 V of COCC structure with: (**a**) EOEC, (**b**) EOCC, and (**c**) COEC structures.

The performance parameters of available gain per unit value of power dissipation, quality factor (g_m_/I_DS_)^35^, and intrinsic gain (g_m_R_O_)^30^ of the device as a function of V_GS_ at V_DS_ = 0.3 V are shown in Fig. 13. Since, g_m_ and g_D_ increase with increasing V_GS_, therefore with a decrease in channel MD and T_OX_ of EOEC and EOCC structures show a significant increase in g_m_/I_DS_ and g_m_R_O_ characteristics. On the other hand, g_m_ and g_D_ of COEC structure decreases when channel MD decreases from 14 to 10 nm whereas when channel MD decreases from 20 to 14 nm causes an increase in g_m_ and g_D_ of COEC structure therefore, g_m_/I_DS_ and g_m_R_O_ first decrease from 20 to 14 nm channel MD then it increases from 14 to 10 nm channel MD for COEC structure.

For g_m_/I_DS_ in the subthreshold region, the transconductance and the drain currents for both variations are negligible. However, the peak of the g_m_ depicts the threshold voltage of the device. From Eq. (2), the drain conductance of the device depends on the tunneling current of TFET^33^. Therefore, increasing the tunneling carrier at the source-to-channel junction causes g_D_ of the device to increase, which consequently leads to the reduction of output resistance (R_O_) of the device. It is observed from Fig. 13a that the optimized EOEC structure shows an increment in g_m_/I_DS_ from ~ 86 V^−1^ to ~ 117 V^−1^ and an increment in g_m_R_o_ from ~ 33 to ~ 90 respectively when compared to COCC structure. Moreover, Fig. 13b depicts, the increment in g_m_/I_DS_ of optimized EOCC from ~ 86 to ~ 96 V^−1^ and increment in g_m_R_o_ from ~ 33 to ~ 79 respectively when compared to COCC structure. Finally, Fig. 13c depicts the COEC structure where g_m_/I_DS_ and g_m_R_o_ first decreases from ~ 86 V^−1^ and ~ 33 (MD = 20 nm) to ~ 78 V^−1^ and ~ 21 (MD = 14 nm) then g_m_/I_DS_ and g_m_R_o_ increases to ~ 79 V^−1^ and ~ 24 respectively. The insertion in Fig. 13c shows the optimized COEC structure having g_m_/I_DS_ and g_m_R_o_ as ~ 81 V^−1^ and ~ 27 respectively.

### Effect of process variation on RF performance parameter

The comparison of variations on EOEC, EOCC, and COEC from its equivalent COCC structure has substantially boosted the RF performance parameters. The performance variation of C_GD_, C_GS_, and C_GG_, along with cut-off frequency (f_T_) and gate delay (τ_m_) is analyzed in this section.

For a fixed V_DS_, the depletion region at the source-to-channel region is formed due to reverse biasing of the source-to-channel junction, which gives rise to C_GS_^36^. However, at low V_GS_, the depletion charge near the drain side decreases with a decrease in the area of the channel, which causes a decrease in C_GD_. When high V_GS_ is applied, the channel area decreases, causing a negligible change in inversion charge at the source end. As a result, C_GS_ decreases with a decrease in the area of the channel. Whereas, the inversion charge increases with a decrease in the area of the channel at high V_GS_. Therefore, C_GD_ increases with a decrease in the area of the channel^37^.

**Figure 14:**
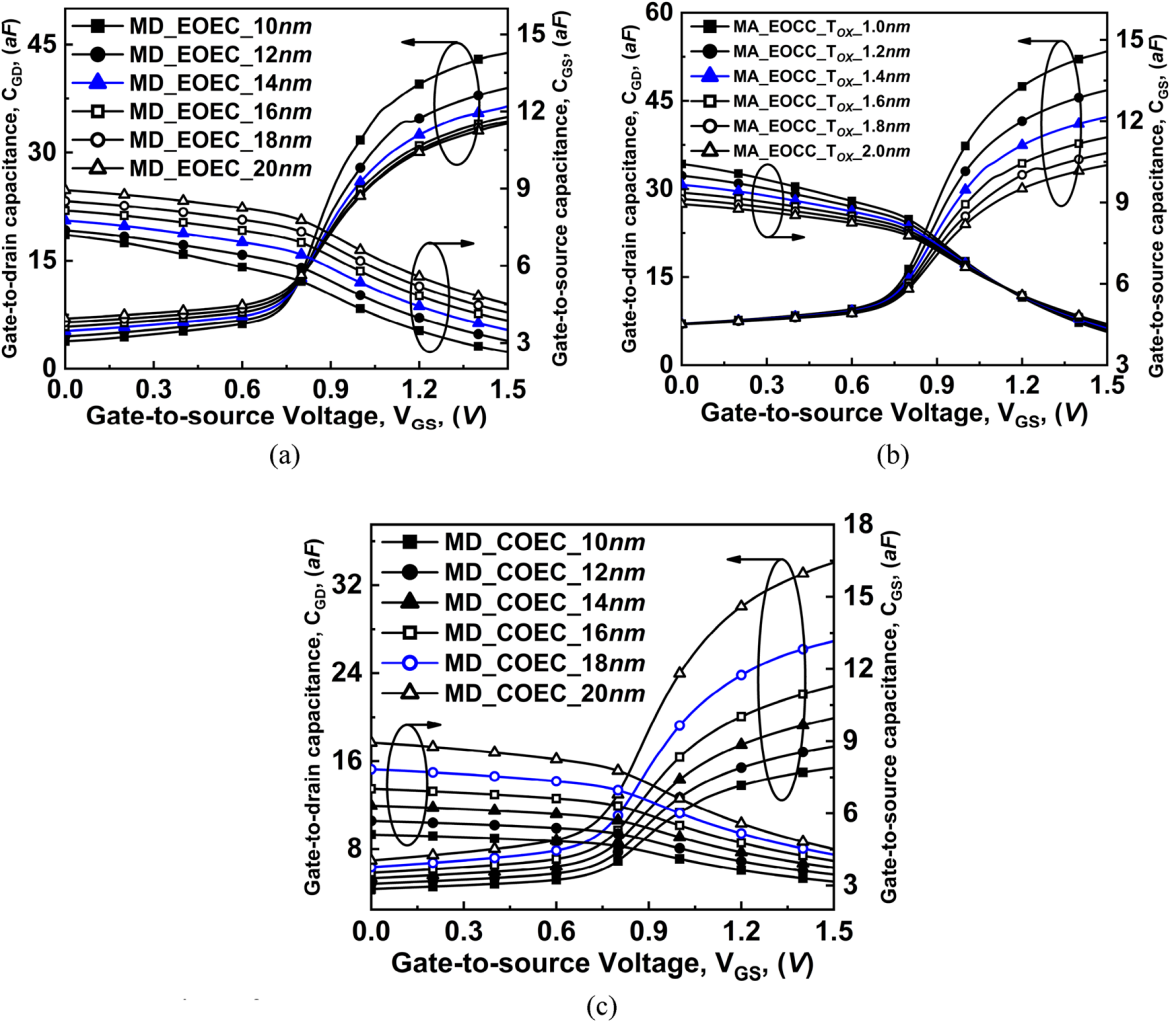
Comparison of variation of gate-to-source capacitances and gate-to-drain capacitances as a function of gate voltage at V_DS_ = 0.3 V of COCC structure with (**a**) EOEC, (**b**) EOCC, and (**c**) COEC structures.

The variation of C_GS_ along with C_GD_ as a function of V_GS_ for EOEC, EOCC, and COEC structures is plotted in Fig. 14. For a fixed V_DS_, when channel MD of the EOEC structure decreases from 20 to 10 nm, it causes a decrease in the area of the GAA structure, which consequently reduces the depletion charge at the source-to-channel junction at low V_GS_. Whereas, at high V_GS_, a decrease in channel MD shows negligible variation in the inversion charge at the source end. As a result, C_GS_ decreases with the decrease in channel MD of the EOEC structure. The optimized EOEC structure shows a decrease in C_GS_ from ~ 4.5 to ~ 3.5 aF when compared to the COCC structure. On the other hand, a decrease in channel MD of the EOEC structure at low V_GS_ causes a negligible change in depletion charge at the drain-to-channel region. Therefore, C_GD_ follows the same trend with a decrease in channel MD of the EOEC structure. However, at high V_GS_, inversion charge experiences a sudden increase therefore, C_GD_ experiences cross-over and increases with a decrease in channel MD of EOEC structure from ~ 34 to ~ 37 aF when compared to COCC structure as depicted in Fig. 14a.

For the EOCC structure, at low V_GS_, decreasing the T_OX_ along MA shows a negligible change in its inversion charge at the source end. As a result, C_GS_ shows negligible variation at low V_GS_. On the other hand, at high V_GS_, decreasing T_OX_ causes a gradual increment in the electric field which subsequently increases the inversion charge near the drain end. Thus, C_GD_ increases with decreasing T_OX_ for EOCC structure which leads to an increase in C_GD_ from ~ 34 to ~ 43 aF when compared to its COCC structure as depicted in Fig. 14b.

On the contrary, unlike the analog performance parameter, the RF performance of COEC follows the predictable trend due to its dependency on the area and T_OX_ of the GAA structure. As the reason stated, the source side depletion charge at low V_GS_ increases with decreasing channel MD of COEC structure, however it experiences slight reduction with virtual increment in T_OX_ where depletion charge at source end is dominated by the variation of channel MD of COEC structure. Furthermore, at high V_GS_, the inversion charge experiences a slight increment at the source side with decreasing channel MD. Therefore, C_GS_ is dominated and decreases with decreasing channel MD of COEC structure. The optimized COEC structure shows a decrement in C_GS_ from ~ 4.5 to ~ 4.2 aF when compared to COCC structure. As channel MD of COEC is dominated by virtual variation of T_OX,_ therefore, at high V_GS_, C_GD_ experiences sudden decrement in inversion charge when compared to its circular structure with a decrease in C_GD_ from ~ 34 to ~ 27 aF from COCC structure as depicted in Fig. 14c.

**Figure 15:**
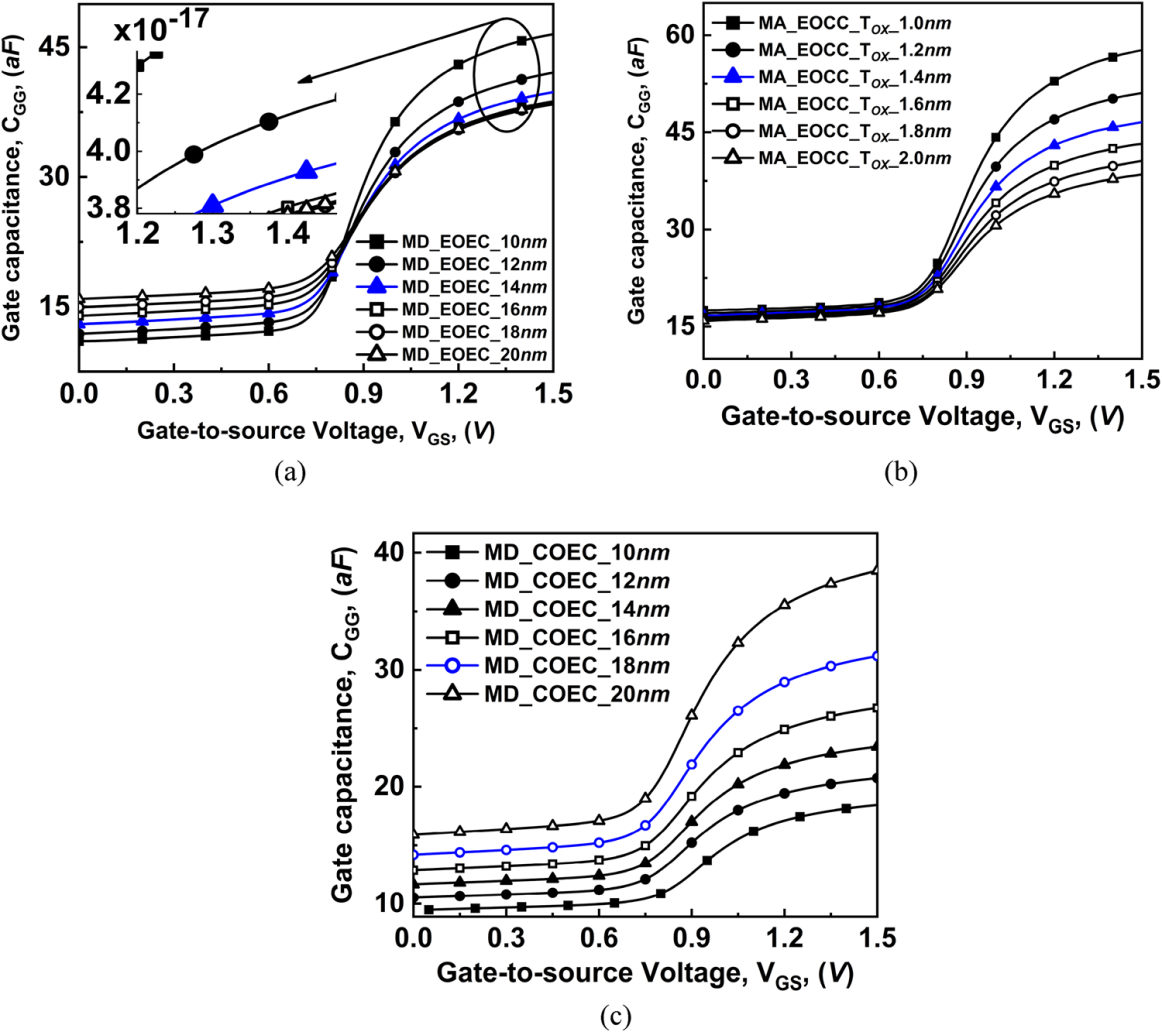
Comparison of variation of total gate capacitance as a function of gate voltage at V_DS_ = 0.3 V of COCC structure with: (**a**) EOEC, (**b**) EOCC, and (**c**) COEC structures.

The total capacitance *C*_*GG*_ = *C*_*GS*_ + *C*_*GD*_ $\left({\mathrm{C}}_{\mathrm{GG}}={\mathrm{C}}_{\mathrm{GS}}+{\mathrm{C}}_{\mathrm{GD}}\right)$ is plotted in Fig. 15 which follows the variation in C_GD_ as depicted in Fig. 14. It is evident from Fig. 15 that, C_GG_ follows the C_GD_ graph which is the dominant contributor to total gate capacitance for any variation in COCC, EOEC, EOCC, and COEC structures. At high V_GS_, C_GG_ is almost the same as C_GD_ whereas, at low V_GS_, C_GG_ shows the same trend as C_GS_ which is higher in magnitude at low V_GS_.

The cutoff frequency (f_T_) and the gate delay (τ_m_) are other parameters for the analysis of RF performances. The cutoff frequency and the gate delay are defined by

3 $$f_{T} = \frac{{g_{m} }}{{2\pi (C_{GS} + C_{GD} )}}$$

4 $$\tau_{m} = \frac{{C_{GG} V_{DD} }}{{I_{ON} }}$$

**Figure 16:**
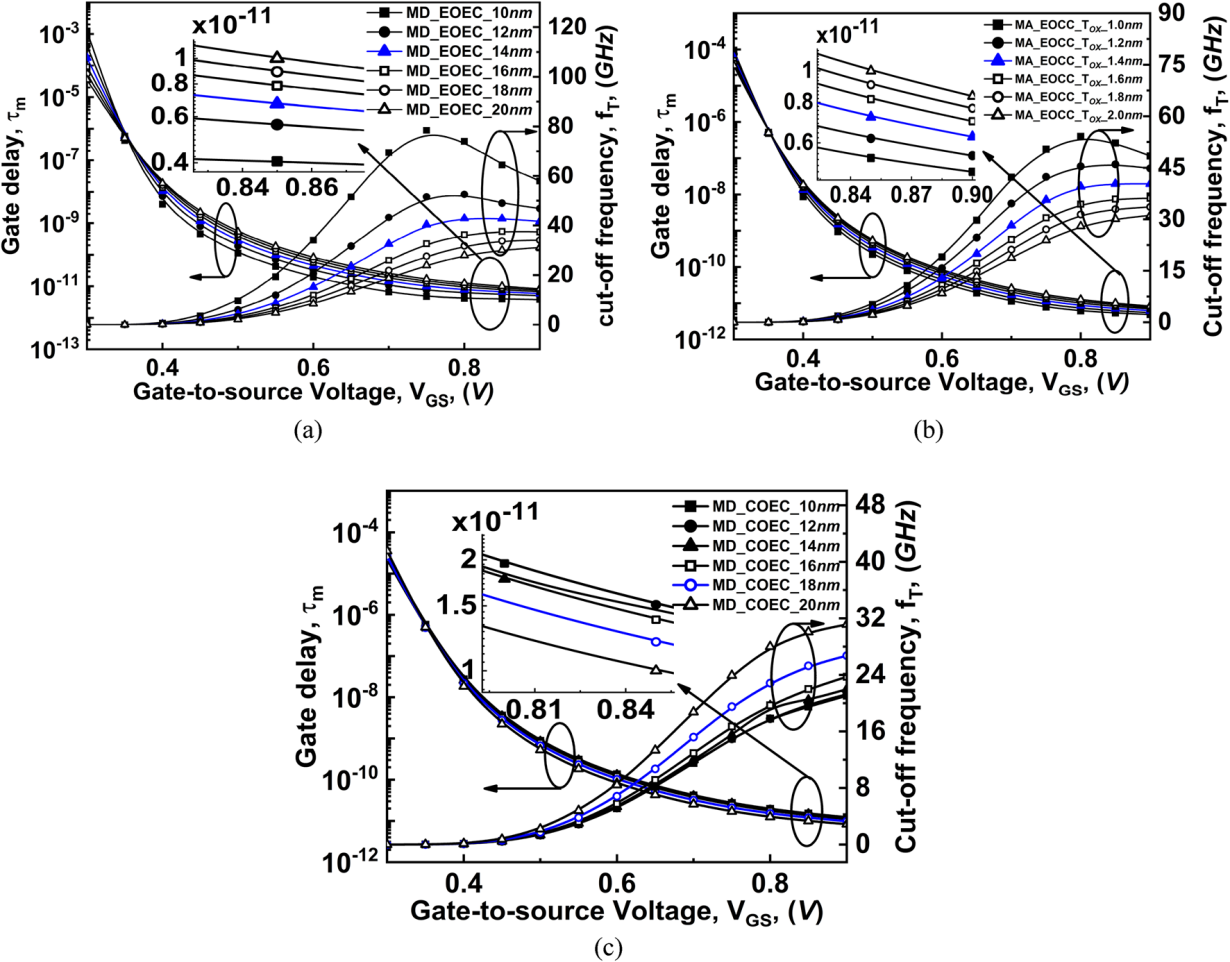
Comparison of variation of cut-off frequency and gate delay as a function of gate voltage at V_DS_ = 0.3 V of COCC structure with: (**a**) EOEC, (**b**) EOCC, and (**c**) COEC structures.

From the f_T_ expression (3), it is evident that it depends on the transconductance (g_m_) and the total capacitance *C*_*GG*_ = *C*_*GS*_ + *C*_*GD*_ values, which is a function of V_GS_^38^. The characteristics of f_T_ are explained explicitly for the subthreshold and superthreshold regions for all device structures. It is observed from Fig. 15 that in the weak inversion region, for each variation, the value of the intrinsic capacitance is low. However, the value of g_m_ increases rapidly in the weak inversion region for variation in any device structure. Therefore, the value of f_T_ at a low V_GS_ value is dominated by the value of g_m_ as shown in Fig. 16. Whereas, in the strong inversion region, the intrinsic capacitance value shows a rapid increase with V_GS_. However, the g_m_ of the device is almost invariable at the strong inversion region. Thus, in strong inversion, the f_T_ parameter is dominated by the value of intrinsic capacitance. Results of the simulation depicted in Fig. 16a that, with the decrease in the channel MD of the EOEC structure, the f_T_ of the device increases where the optimized EOEC structure shows increment from ~ 31 to ~ 43 GHz cutoff frequency and increment from ~ 31 to ~ 40 GHz cutoff frequency on EOCC structure when compared to COCC structure. On the other hand, for the COEC structure, intrinsic capacitance is relatively higher. Therefore, the value of f_T_ at a low V_GS_ value has an impact on both the intrinsic capacitance and the g_m_ of the device. As a result, the decrease in MD of the COEC structure causes a decrease in f_T_ of the device where the optimized COEC structure shows a decrement from ~ 31 to ~ 26 GHz when compared to its circular structure as shown in Fig. 16c.

The variation of gate delay (τ_m_) as a function of V_GS_ is also plotted in Fig. 16. The gate delay expression (4) stated earlier has supply voltage (V_DD_), gate capacitance (C_GG_), and ON-state current (I_*ON*_) dependence.

It is observed from Fig. 15a that, with a decrease in channel MD of EOEC structure causes C_GG_ and drain current to increase following the reason stated in analog performance. The rate of increase of drain current is dominated by the increase in C_GG_ of the device, which consequently reduces the τ_m_. The insertion in Fig. 16a depicts the decrease in the τ_m_ of optimized EOEC structure from ~ 8.5 to ~ 5.5 psec when compared to COCC structure. For EOCC structure, shows an increment in both C_GG_ as well as drain current with decreasing T_OX_ of the device. Likewise, considering the case of EOEC, the drain current of the EOCC structure shows dominating parameter when compared to the C_GG_ of the device followed by a reduction in τ_m_ as depicted in Fig. 16b. The enlarged view under Fig. 16b shows the reduction of optimized gate delay from ~ 8.5 to ~ 6.3 psec when compared to its circular structure. Finally, for the COEC structure, variation in channel MD causes virtual variation in T_OX_ which in turn reduces C_GG_ and drain current of the device. Likewise, the drain current shows dominating nature when compared to C_GG_ which further led to an increase in τ_m_ of the device as depicted in Fig. 16c. The insertion in Fig. 16c shows the optimized COEC gate delay from ~ 8.5 to ~ 10 psec when compared to the COCC structure.

### Benchmarking of proposed EOEC and COCC structures against several GAA MOSFETs and TFETs reports

This section presents a status map of numerous reports available on elliptical or circular GAA MOSFETs and TFETs against the analyzed EOEC and COCC structures as tabulated in Table 2. The status map shows that there is a drastic decrease in I_*ON*_ for GAA TFET when compared to its GAA MOSFET counterpart. This drastic decrease is due to different carrier injection mechanisms as well as comparatively lower V_DS_ applied to the device. On the other hand, the device under consideration also has a lower I_*ON*_ when compared to its TFET counterpart. This lower I_*ON*_ is due to the lower V_DS_ applied to the device under consideration. However, the device shows significantly reduced gate capacitance, which is the crucial parameter for any device’s performance. Besides the lower I_*ON*_, the device under consideration shows a drastic increase in the I_*ON*_/I_*OFF*_ ratio. This high I_*ON*_/I_*OFF*_ determines the switching speed of the device.”

**Table 2:** Details of proposed EOEC and COCC structures against several GAA MOSFETs and TFETs.

Geometry	This work		Ref.^12^	Ref.^13^	Ref.^15^	Ref.^14^	Ref.^9^	Ref.^39^	Ref.^40^
	COCC TFET	EOEC TFET	EOEC MOSFET	EOEC MOSFET	EOEC MOSFET	COCC MOSFET	COCC TFET	DG TFET	COCC TFET
Diameter (nm)	20	14	~ 20.4	~ 12.8	~ 13.9	~ 3	10	–	20
L_G_ (nm)	30	30	~ 35	~ 25	~ 25	10	20	100	50
EOT or T_OX_ (nm)	2	2	1.5	~ 1.12	~ 1.4	1	2	2	2
V_DS_ (V)	0.3	0.3	1	1	1	0.4	1	1	1
I_*ON*_ (μA/μm)	~ 32	~ 44	825	976	600	~ 1180	244	148	200
I_*ON*_/I_*OFF*_	3.4 × 10^13^	1.9 × 10^11^	2 × 10^5^	9.7 × 10^5^	6 × 10^7^	1.4 × 10^5^	1.3 × 10^8^	1.8 × 10^11^	7.12 × 10^12^
g_m_ (mS)	~ 0.051	~ 0.069	–	–	–	–	–	0.1	0.55
C_GG_ (fF)	0.041	0.037	–	–	–	–	–	1.1	2.5
f_T_ (GHz)	32	44	–	–	–	–	–	–	40

## Conclusion

The optimization of the structure and the comparative analysis of analog and RF FOMs for the III-V eGAA TFET with its EOEC, EOCC, and COEC structures are performed. Further optimization and comparative analysis of the analog and RF FOMs of the device is done for various EOEC, EOCC, and COEC structures. The device shows comparatively higher mobility, lower parasitic capacitances, and a minimum possible gate delay of the optimized geometries. When compared to their circular structures, the optimised EOEC and EOCC devices show a 64% and 59% increase in intrinsic gain, respectively, and a 28% and 23% increase in cut-off frequency. On the other hand, the optimized COEC structure shows a reduction in its intrinsic gain and its cut-off frequency. However, the physics behind the extracted output characteristics depends upon the dominancy of T_OX_ and MD of the COEC structure. Owing to the higher gain and cut-off frequency of the EOEC and EOCC structures, the proposed optimized device can be an alternative structure that meets the need for higher performance for analog RF CMOS circuit applications. To summarize, the advantage of the undesired fabrication issue up to a certain level (MD up to 30% for EOEC, T_OX_ up to 30% for EOCC, and MD up to 10% for COEC structures), which enhances the analog and RF FOMs, can be passed on to the circuit design where high gain, higher drive current, and higher gain bandwidth product are desired, and thus, the unintentional elliptical device structure proves to be the promising choice for analog/RF application.

## Data Availability

The datasets generated and/or analyzed during the current study are available from the author Pankaj Kumar (iitdhn.pankaj@gmail.com) upon reasonable request.
